# ON-NSW: Accelerating High-Dimensional Vector Search on Edge Devices with GPU-Optimized NSW

**DOI:** 10.3390/s25206461

**Published:** 2025-10-19

**Authors:** Taeyoon Park, Haena Lee, Yedam Na, Wook-Hee Kim

**Affiliations:** Department of Computer Science and Engineering, Konkuk University, Seoul 05029, Republic of Korea

**Keywords:** industrial internet of things (IIoT), on-device AI, edge computing, vector database, approximate nearest neighbor search (ANNS), GPU acceleration

## Abstract

The Industrial Internet of Things (IIoT) increasingly relies on vector embeddings for analytics and AI-driven applications such as anomaly detection, predictive maintenance, and sensor fusion. Efficient approximate nearest neighbor search (ANNS) is essential for these workloads. Graph-based methods are among the most representative methods for ANNS. However, most existing graph-based methods, such as Hierarchical Navigable Small World (HNSW), are designed for CPU execution on high-end servers and give little consideration to the unique characteristics of edge devices. In this work, we present ON-NSW, a GPU-optimized design of HNSW optimized for edge devices. ON-NSW employs a flat graph structure derived from HNSW to fully exploit GPU parallelism. In addition, it carefully places HNSW components in the unified memory architecture of NVIDIA Jetson Orin Nano. Also, ON-NSW introduces warp-level parallel neighbor exploration and lightweight synchronization to reduce search latency. Our experimental results on real-world high-dimensional datasets show that ON-NSW achieves up to 1.44× higher throughput than the original HNSW on the NVIDIA Jetson device while maintaining comparable recall. These results demonstrate that ON-NSW provides an effective design for enabling efficient and high-throughput vector search on embedded edge platforms.

## 1. Introduction

In the Internet-of-Things (IoT) era, the amount of sensor data has increased dramatically [1]. In particular, industries deploy various sensors to enable more efficient manufacturing processes. The sensor data is highly informative for industrial applications such as predictive maintenance [2,3], anomaly detection [4,5], and intelligent automation [6]. For example, in predictive maintenance, sensor data collected from turbofan engines are analyzed to estimate the remaining useful life of equipment and schedule maintenance at appropriate times. In anomaly detection, time-series data such as heartbeat and engine signals are analyzed to identify abnormal patterns in operational behavior. In intelligent automation, sensor data including temperature and vibration from smart manufacturing environments are leveraged to autonomously adjust operational parameters and maintenance schedules in real time.

To effectively leverage the sensor data, industries have been relying on real-time analysis and retrieval, where low latency and high throughput are critical [7]. Typically, the sensor data is transferred to the cloud, where they are processed for further analysis. However, this cloud-based approach often suffers from performance degradation due to the communication overhead. Moreover, because this approach depends heavily on data transfer, it can also raise privacy concerns.

To address these limitations of the cloud-based approach, on-device processing for the Industrial Internet of Things (IIoT) offers promising alternatives [8,9,10]. With on-device processing, real-time analysis and retrieval can be performed without transferring data to the cloud, thereby reducing latency and alleviating privacy concerns [11]. In addition, advances in embedded hardware platforms, such as NVIDIA Jetson devices (NVIDIA Corporation, Santa Clara, CA, USA), provide powerful GPU resources that enable efficient execution of machine learning and search algorithms at the network edge.

Among various analytics tasks in IIoT, Approximate Nearest Neighbor Search (ANNS) has emerged as a fundamental operation for processing high-dimensional feature embeddings derived from sensor data [12]. ANNS enables efficient similarity search, which is critical for industrial applications [13,14]. Existing ANNS algorithms can be broadly categorized into three groups [15]: (1) tree-based, (2) hashing-based, and (3) graph-based approaches. Tree-based methods, such as KD-Tree [16], provide good performance for low-dimensional vector data, but suffer performance degradation when the dimension increases. Hashing-based methods highly depend on hash functions. Among these, graph-based approaches, such as NSW [17] and HNSW [18] are widely adopted because they support dynamic insertions and deletions while also achieving high efficiency in nearest neighbor searches.

However, most ANNS algorithms were originally designed for high-performance servers, making them difficult to deploy directly on edge devices. Furthermore, edge devices exhibit unique architectural characteristics, such as a limited number of CPU cores and weaker computing power compared to servers [19]. In addition, CPUs and GPUs share the same memory space through a unified architecture. These characteristics require tailored algorithmic designs to fully exploit the capabilities of edge devices.

In this work, we propose ON-NSW, a graph-based ANNS algorithm specifically tailored for NVIDIA Jetson devices, a representative platform for edge computing. ON-NSW is designed to accommodate the unique GPU memory hierarchy of edge devices and fully exploit GPU resources to achieve high-performance ANNS. Experimental results demonstrate that ON-NSW achieves up to 1.44× higher throughput than CPU-optimized algorithms on high-dimensional datasets. The contributions of this paper are as follows:

- We propose a GPU-centric NSW design that removes the upper hierarchy of HNSW and executes the entire bottom-layer search on the GPU for on-device ANN tasks.
- We design ON-NSW, which exploits the NVIDIA Jetson device’s unified architecture by enabling zero-copy access to pinned memory and maximizes efficiency during search by leveraging GPU shared memory.
- We implement parallel neighbor exploration that leverages GPU cores to concurrently fetch neighbors and compute distances for efficient search.

The remainder of this paper is organized as follows. Section 2 provides the background of this work, and Section 3 elaborates on the design. Section 4 provides the experimental results. Section 5 discusses related work, and Section 6 concludes the paper.

## 2. Background

In this section, we briefly describe the background and fundamental concepts relevant to this work. We first introduce Approximate Nearest Neighbor Search (ANNS) and its main algorithmic categories. After that, we explain the Hierarchical Navigable Small World (HNSW) that serves as the basis of our design. We then discuss the GPU architecture of the NVIDIA Jetson device to provide the necessary context for our GPU-optimized ON-NSW algorithm.

### 2.1. Approximate Nearest Neighbor Search (ANNS)

Unstructured data such as images and videos are managed by converting them into high-dimensional vector data using an AI model. In large-scale high-dimensional datasets, finding vector data similar to a query is crucial because it directly impacts the performance of various applications (e.g., recommendation systems, image or video retrieval, and anomaly detection). The k-Nearest Neighbor Search (k-NNS) algorithm addresses this challenge. It computes the distances between the query vector and all vectors in the database to return the top-k vectors closest to the query [20]. While it guarantees accurate results, the cost of computing distances becomes extremely high as the data size and dimensionality increase, making it impractical for real-time search. To overcome this limitation, Approximate Nearest Neighbor Search (ANNS) trades a small amount of accuracy for higher computational efficiency [21]. Instead of identifying the exact nearest neighbors, ANNS aims to retrieve the approximately nearest neighbors. The quality of the search results is evaluated by recall, which measures the proportion of true nearest neighbors included in the search results.

ANNS algorithms can be categorized into three groups: tree-based, hashing-based, and graph-based algorithms. Tree-based ANNS (e.g., KD-tree [16], Ball tree [22]) reduces the search space by partitioning data into a tree structure. While this approach is highly effective for low-dimensional data, its efficiency rapidly decreases as dimensionality increases. Hashing-based ANNS (e.g., LSH [23], Multi-Probe LSH [24]) maps data into hash buckets so that similar vector data is stored in the same bucket. This enables very fast search, but makes accuracy highly dependent on the design of the hash function. Graph-based ANNS (e.g., NSW [17], HNSW [18]) constructs a graph where data points are nodes and their similarities are represented by edges. This approach provides both high accuracy and fast search performance, and it remains robust even in high-dimensional data. Among these categories, graph-based algorithms have been most actively studied [25,26] and are now widely adopted across various industries [27,28].

### 2.2. Hierarchical Navigable Small World (HNSW)

Navigable Small World (NSW) [17] is a fundamental graph-based approach that constructs a single graph where data points are represented as nodes and their similarity is represented as edges. The search process begins at an entry point and greedily traverses toward closer neighbors. However, as the dataset grows, the search paths become longer, and the performance becomes highly dependent on the choice of entry point.

Hierarchical Navigable Small World (HNSW) [18] addresses these issues by introducing a hierarchical structure into the graph. When data is inserted, it is assigned to multiple layers up to a randomly selected maximum level. Upper layers are sparsely connected, enabling the search to quickly approach the query, while lower layers are densely connected, enabling more precise searches. Toward the lower layers, the number of nodes grows logarithmically, and the lowest layer (layer 0) contains all the data. The search begins at the topmost layer and progressively proceeds downward, as illustrated in Algorithm 1. In the sparse upper layers, the algorithm traverses over distant nodes and identifies the node closest to the query within that layer, which then serves as the entry point to the next layer. By repeating this process, the search algorithm can identify neighbors progressively closer to the query. This hierarchical design shortens search paths and provides higher accuracy compared to NSW. Although HNSW provides efficient ANNS on high-end server CPUs, deploying such algorithms on edge devices introduces new challenges due to limited computing resources and distinct memory hierarchies. In the following section, we describe the GPU and memory structure of NVIDIA Jetson devices, which serve as representative edge platforms.
**Algorithm 1:** Search algorithm of HNSW [18]
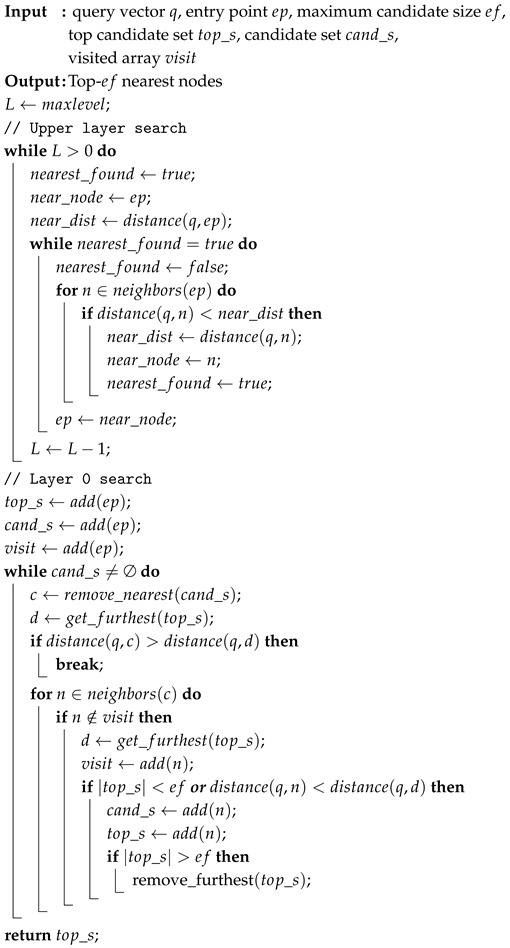


### 2.3. GPU Structure on NVIDIA Jetson Device

The NVIDIA Jetson device is a representative edge device equipped with a GPU [29]. This device consists of two sockets, each containing three Cortex-A78AE ARMv8.2 cores, (ARM Ltd., Cambridge, UK). Each core has a 64 KB L1 cache and a 256 KB L2 cache, and each socket includes a 2 MB L3 cache. The system is connected to 8 GB of 128-bit LPDDR5 DRAM. The GPU is based on the Ampere architecture and contains 1024 CUDA cores. It is composed of eight Streaming Multiprocessors (SMs), with each SM capable of maintaining up to 1536 threads concurrently. Each SM is equipped with on-chip SRAM of 164 KB, which is shared between the L1 cache and the shared memory. The entire GPU shares a 2 MB L2 cache.

**Figure 1:**
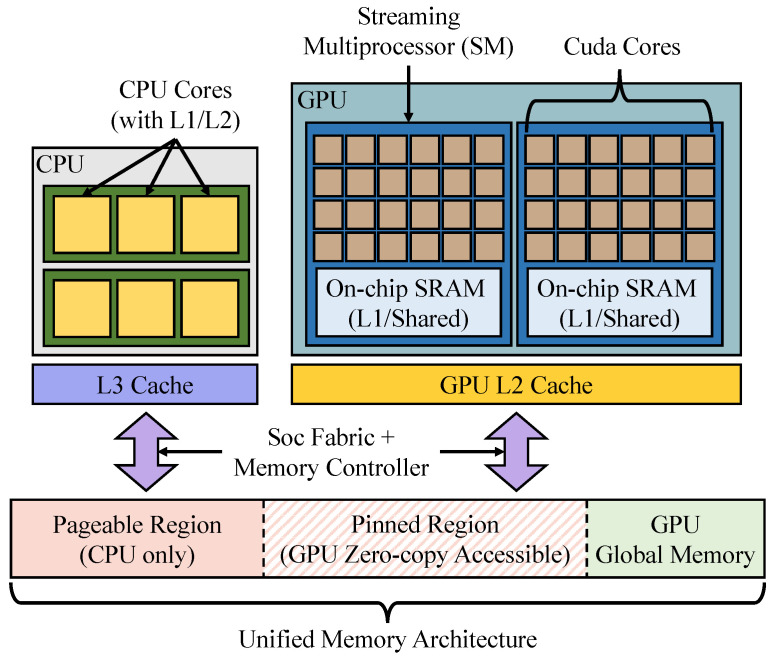
Memory hierarchy of the NVIDIA Jetson device, illustrating the CPU-GPU memory hierarchy and the placement of pinned memory.

As shown in Figure 1, unlike general server environments equipped with discrete GPUs, NVIDIA Jetson device adopts a unified memory architecture in which the GPU global memory and CPU host memory reside in the same DRAM. Therefore, both GPU and CPU can directly access the same DRAM space through the SoC fabric and the memory controller. Under this architecture, the overhead of copying data from host (CPU) to device (GPU), as required in server environments, is eliminated. Moreover, the GPU can perform direct memory access (DMA) to pinned regions of host memory in a zero-copy manner. In server environments, DMA to pinned regions is carried out by PCIe, which incurs relatively higher latency, whereas the NVIDIA Jetson device avoids this overhead.

However, because the operating system forces pinned memory pages to remain in a page-locked state, CPU caching policies are limited. As a result, accessing pinned memory can incur higher latency for the CPU compared to pageable memory. From the GPU perspective, accessing pinned memory also incurs higher latency than GPU global memory access due to additional costs for cache coherence management. However, it still avoids the overhead of explicit data copies, making it more efficient than the copy-based method.

The original HNSW was designed for high-end CPUs and discrete GPUs, and therefore does not fully exploit the architectural characteristics of edge devices. In particular, NVIDIA Jetson devices employ a unified memory hierarchy in which CPUs and GPUs share the same DRAM space [30]. This feature requires careful design of data placement and memory access policies to fully exploit the device’s performance. These limitations of the original HNSW motivate our GPU-optimized redesign for edge devices, as described in the following section.

## 3. Design

In this section, we present the design of ON-NSW, which is optimized for edge devices. We begin with an overview of the system architecture and then elaborate on the core components and mechanisms of ON-NSW.

### 3.1. Design Overview

ON-NSW employs a GPU-optimized design tailored for edge devices such as the NVIDIA Jetson device. The main idea is to restructure the original HNSW in a way that maximizes GPU parallelism while reducing data access latency. To this end, ON-NSW flattens the hierarchical graph and retains only the bottom layer (layer 0), enabling the entire search operation to run directly on the GPU, as illustrated in Algorithm 2. This design allows multiple candidate nodes to be explored simultaneously, while their distances to the query vector are computed in parallel. Furthermore, ON-NSW optimizes memory placement to exploit NVIDIA Jetson device’s unified CPU-GPU architecture.
**Algorithm 2:** Search algorithm of ON-NSW
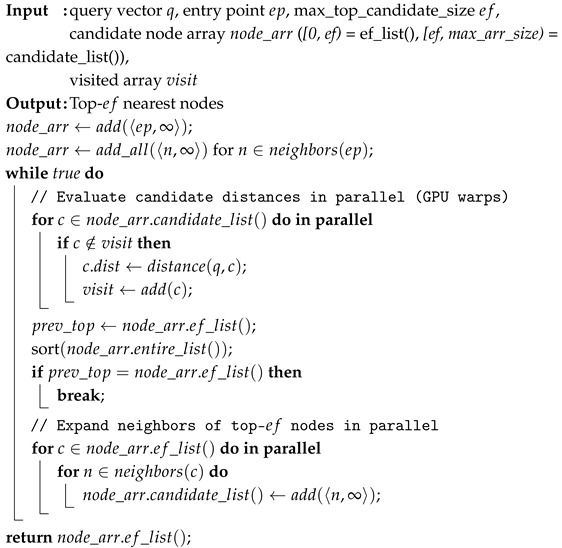


ON-NSW contains the following components: vector data, visited arrays, top-k results, query vectors, and candidate arrays. Data shared between CPU and GPU, including vector data, visited arrays, and top-k results, are stored in pinned memory to avoid copy overhead. In contrast, query vectors are placed in GPU global memory and candidate arrays are allocated in shared memory to reduce latency. ON-NSW also leverages warp-level parallelism for neighbor exploration, where distances to multiple candidates are computed concurrently and neighbors are expanded in parallel. This approach enables ON-NSW to visit more nodes per hop, thereby accelerating convergence to the nearest neighbors.

### 3.2. Flat Graph Structure for GPU Parallelism

HNSW was originally designed to improve search efficiency by leveraging a hierarchical structure. The algorithm first traverses the upper layers to move closer to the query vector, and once it reaches layer 0, it identifies the *top-k* approximately nearest neighbor nodes. Starting the search directly from the entry point in layer 0 would require visiting many nodes before reaching the query, since layer 0 contains all data points. By traversing the upper layers, HNSW reduces search overhead by skipping a large portion of nodes in layer 0.

This approach is effective in CPU-only environments, as the original HNSW explores neighbors sequentially. However, on GPUs, the upper-layer search can become a performance bottleneck, since the operations in these layers limit GPU parallelism. As each layer’s search is tightly dependent on the previous one, it is difficult to parallelize the process on GPUs. A hybrid approach combining CPU and GPU could be considered, where the upper-layer search is performed on the CPU. However, this approach does not significantly reduce the search overhead in layer 0. In addition, sharing the same memory region between CPU and GPU requires the pinned memory, which incurs higher latency than pageable host memory.

To address these limitations, ON-NSW adopts a flat, single-layer structure in which layer 0 contains the entire vector dataset without additional upper layers, thereby maximizing GPU parallelism and reducing memory access overhead. In ON-NSW, the GPU leverages its CUDA cores to simultaneously compute distances between the query vector and multiple nodes. This design enables the search to reach nodes closer to the query more quickly than the hierarchical structure of the original HNSW. Unlike CPUs, where sequential traversal can benefit from branch prediction and caching, GPUs achieve greater efficiency when all nodes are organized within a single layer that fully exposes parallelism. Moreover, maintaining a flat structure simplifies memory management and allows for a more systematic placement of data across different memory regions, which we further optimize in Section 3.3. Although the search in ON-NSW begins from a fixed entry point and thus requires more hops to approach the query, GPU parallelism allows distances to be computed for multiple nodes and candidate nodes to be expanded simultaneously at each hop, thereby reducing the overall search time.

Unlike the hierarchical HNSW with logarithmic search complexity, ON-NSW’s single-layer structure has linear theoretical complexity O(N). However, its GPU-based parallel execution effectively mitigates this overhead and yields faster practical performance on high-dimensional datasets.

### 3.3. Optimizing Data Placement in a Unified Memory Architecture

**Figure 2:**
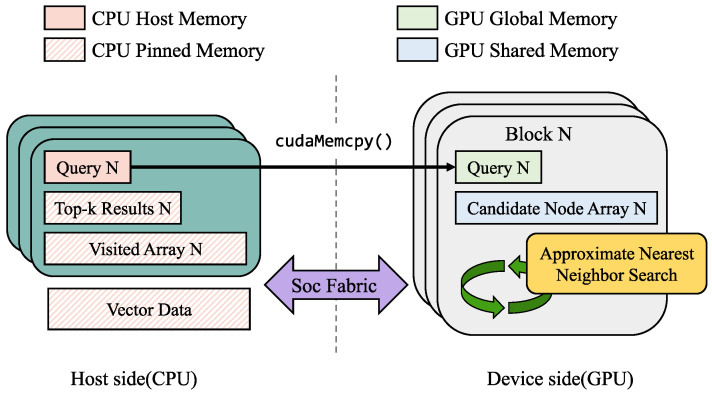
Overview of the GPU memory hierarchy and the placement of ON-NSW. The gray boxes on the right denote GPU blocks, while the green boxes on the left indicate that each block maintains its own query, top-k result array, and visited array.

Edge devices such as the NVIDIA Jetson device are based on a System-on-Chip (SoC) architecture. In these SoC platforms, the CPU’s host memory and the GPU’s global memory share the same physical DRAM space and are accessed through the SoC fabric. This structure makes the memory hierarchy of edge devices more complicated. To fully exploit the device’s potential, ON-NSW carefully places each component in the appropriate memory region, considering the architectural characteristics, as illustrated in Figure 2.

**Vector Data.** ON-NSW stores vector data in pinned memory, which is accessible to both the CPU and GPU. The GPU can directly access pinned memory through the SoC fabric using DMA, enabling zero-copy data transfer between the host and GPU. However, because pinned pages are locked in physical memory, they cannot take advantage of CPU-side caching policies and may therefore incur higher latency for CPU access compared to pageable memory.
**Visited Array.** During the search process, the visit state of each node is tracked using a visited array. The visited state indicates that the distance to a node has already been calculated, thereby preventing redundant distance calculations for the same node. Since this array must have the same size as the number of data points in the dataset, it is too large to fit in GPU shared memory and is therefore placed in pinned memory.
**Top-k Results.** As the top-k results found by the GPU must be accessible by both CPU and GPU, ON-NSW stores the top-k result array in pinned memory. This placement allows CPU and GPU to share the results without incurring additional copy overhead.
**Query Vector.** Query vector is used solely during the GPU search process. ON-NSW copies it into GPU global memory and utilizes it within the GPU kernel. To reduce the overhead of cudaMemcpy() calls, ON-NSW processes queries in batches.
**Candidate Array.** The candidate array stores the IDs and distances of nodes whose similarities to the query vector have been calculated. After each iteration, the array is sorted by distance, and the *top-ef* nodes are retained for further neighbor expansion in the next step. If the *top-ef* entries remain unchanged after sorting, the search is considered converged and terminates. Therefore, the candidate array is essential for managing neighbor expansion and determining the termination condition during the GPU-based search.

### 3.4. Parallel Neighbor Exploration at Warp Level

**Figure 3:**
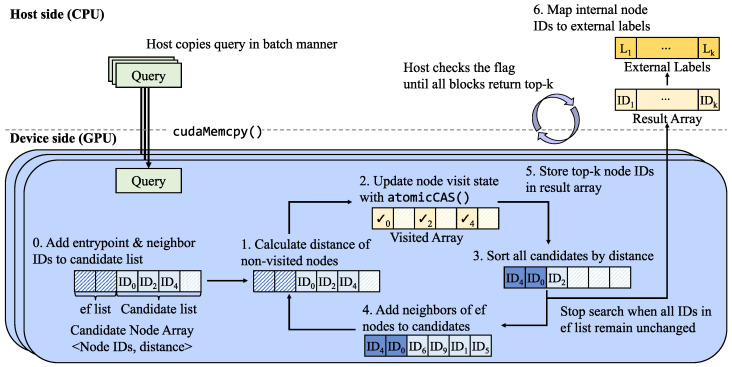
Workflow of the GPU-based search operation in ON-NSW. The shaded entries in the Candidate Node Array indicate empty elements. The bold arrow from CPU to GPU denotes that each query is copied to a GPU block via cudaMemcpy(), whereas the regular arrows represent the process flow of the search operation. Checkmarks indicate that the corresponding nodes have been visited. For clarity, elements of the Result Array and External Labels are partially omitted.

To maximize parallelism on the GPU, ON-NSW leverages warp-level execution for the search operation. Figure 3 illustrates how ON-NSW processes the search operation. In ON-NSW, each GPU block is assigned to handle one query vector during the search. Within a block, the 32 threads of each warp cooperatively compute the distance between the query vector and a candidate node (represented as a vector) by evenly dividing the vector dimensions among threads (e.g., 32 dimensions per thread for a 1024-dimensional vector). Multiple warps within a block process different candidate vectors concurrently, maximizing GPU utilization. The vector data are stored in the pinned region of unified memory. A warp is the basic execution unit of a GPU block, where each warp processes one vector in parallel. This enables ON-NSW to compute the distances of multiple nodes in the candidate array simultaneously. Furthermore, when adding new candidate nodes, ON-NSW inserts the neighbors of the current nearest nodes in parallel, thereby expanding the search space. Unlike the original HNSW, where each CPU thread sequentially processes a single query and visits each node only once, ON-NSW explores the neighbors of all *ef* nodes in parallel at each hop. Consequently, ON-NSW can visit more nodes within a single hop and reach nodes closer to the query faster, thereby improving search efficiency.

As shown in Figure 3, ON-NSW first copies query data in batches to GPU global memory using cudaMemcpy(), and each GPU block processes one query. To coordinate the completion of blocks, ON-NSW employs a lightweight synchronization mechanism based on a shared flag variable, which ensures that all blocks have produced their top-k search results.

The GPU first initializes the candidate array, which consists of two parts: the *ef list*, storing the nearest nodes, and the candidate list, storing additional nodes to be explored. During initialization, each node ID is set to UINT32_MAX and each distance to FLT_MAX. The IDs of the entry point node and its neighbors are then inserted into the candidate list, with their distances initialized to FLT_MAX since they have not yet been computed.

Each warp, composed of 32 threads, is responsible for calculating the distance between the query and one node, while multiple warps operate concurrently to process many nodes in parallel. To reduce unnecessary computation, each GPU thread checks the visited array for its warp and skips the calculation if the node has already been visited. Subsequently, the visited state of each node is updated using the atomicCAS instruction. If the update succeeds, the node is preserved. Otherwise, its entry ID and distance are set to UINT32_MAX and FLT_MAX, indicating that the corresponding node has already been processed by another thread. This approach prevents duplication in the candidate array.

After distance calculations and visited state updates, ON-NSW sorts the candidate array in ascending order of distance and checks node IDs in *ef list*. If the nodes in the *ef list* are updated, the neighbors of its nodes are added to the candidate list. This expansion step is also executed in parallel, where each warp is assigned a node and the threads within the warp cooperatively insert its neighbors into the candidate list.

Otherwise, if the nodes in the *ef list* remain unchanged after sorting, ON-NSW terminates the search and stores the top-k node IDs and their distances from the *ef list* into the result array. Once the results are stored, a synchronization flag is updated using the atomicAdd instruction. Finally, ON-NSW checks whether the flag equals the product of the number of blocks and the top-k size. After this condition is satisfied, ON-NSW maps the node IDs to their external labels provided by users and returns the final search results.

### 3.5. Optimizations

ON-NSW employs several optimizations to enhance search efficiency.

**Flag-based synchronization.** As introduced earlier, ON-NSW uses a synchronization flag to coordinate the completion of GPU blocks. Launching a CUDA kernel typically requires the CPU to wait for completion by invoking cudaDeviceSynchronize(), but we observed that this synchronization method introduces significant overhead. To mitigate this cost, ON-NSW employs a flag-based lightweight synchronization mechanism. Each GPU block updates the flag variable using atomicAdd once it completes its search. The CPU then checks the flag through spin-wait loop until all blocks have finished. This method reduces synchronization overhead and improves overall performance.
**Vectorization.** During the search operation, the most time-consuming operation is distance calculation, particularly for high-dimensional vectors. To optimize this process, ON-NSW vectorizes the floating-point values into float4 type, enabling 128-bit memory loads. This approach ensures coalesced memory access and improves GPU memory bandwidth utilization.
**Prefetching.** To hide the high latency of pinned memory, ON-NSW loads multiple nodes at once during the warp-based distance calculation. This prefetching allows memory access latency to overlap with calculation, leading to enhanced search efficiency.
**Multithreading for Mapping.** After the search, ON-NSW employs multithreading on CPU to map internal node IDs to external labels in parallel, thereby reducing the execution time.

## 4. Evaluation

This section presents the experimental setup and evaluates ON-NSW compared with the original HNSW in terms of search quality, throughput, energy efficiency, and GPU utilization on an edge device.

### 4.1. Experimental Environment

**System Configuration**. We conduct experiments on an NVIDIA Jetson Orin Nano (NVIDIA Corporation, Santa Clara, CA, USA), equipped with two sockets of Arm Cortex-A78AE v8.2 64-bit processors (Arm Ltd., Cambridge, UK), with each socket containing three cores. The system has 8 GB of memory, and the SoC integrates a GPU based on the Ampere architecture. The GPU consists of 8 Streaming Multiprocessors (SMs) and 1024 CUDA cores, with a maximum clock frequency of 625 MHz. Each warp is organized into 32 threads, and a single GPU block can support up to 1024 threads with 48 KB of shared memory. In addition, the GPU global memory is shared with the system DRAM.
**Dataset**. We use 4 real-world datasets: three high-dimensional and one low-dimensional vector-embedding dataset. For the high-dimensional datasets, we use OpenAI [31,32] text embeddings with 1536 and 3072 dimensions as well as the GIST [33] dataset with 960 dimensions. For the low-dimensional case, we use the SIFT [33] dataset with 128 dimensions. All datasets consist of 4-byte floating-point values. We build graph index structures with 50,000 vectors, randomly selected from each original dataset.  

A randomly sampled dataset may exhibit a different distribution from the original dataset. Therefore, to verify whether the sampled dataset retains a distribution similar to the original one, we employ the Fréchet Inception Distance (FID) [34]. FID is a metric commonly used to evaluate the performance of image generation models such as Generative Adversarial Networks (GANs) [35]. It measures the distance between the feature distributions of generated and original images. Because image data are inherently high-dimensional, this property makes FID suitable for assessing the distributional similarity of high-dimensional embedding data. An FID value close to zero indicates that the two datasets share highly similar distributions. We calculated the FID values for all sampled datasets and found that each exhibits a very small value (below 0.01), indicating that the sampled datasets effectively preserve the original data distribution.

We construct the ground truth for each query by computing the distances to all sampled vectors and selecting the top 1000 data IDs. The selected IDs are then sorted in an ascending order based on their distance to the query vector.

**Baseline**. For comparison, we use the original HNSW implementation provided by hnswlib [36] and execute it on the NVIDIA Jetson device. We set the index-building parameters to *M* = 16 and *efConstruction* = 100 for both the original HNSW and ON-NSW. Since layer 0 maintains twice as many neighbors, the value of *M* for layer 0 is set to 32 in both implementations. Following the original HNSW design, layer 0 is configured to have twice as many maximum neighbors as the upper layers. Because layer 0 contains all vector data, maintaining a larger number of connections helps preserve local connectivity among nearby nodes and improves search efficiency. We use the L2 (Euclidean) distance as the similarity metric. In addition, we modified the original distance computation function of HNSW, which was optimized for x86_64 SIMD instructions, to instead use ARM_NEON instructions.

### 4.2. Graph Search Quality

We compare the throughput of the graph index structures with respect to Recall@10. The original HNSW performs searches using six threads, whereas ON-NSW employs 32 GPU blocks, each consisting of 1024 threads. In addition, ON-NSW uses six CPU threads to map internal data IDs to external labels after the search.

**Figure 4:**
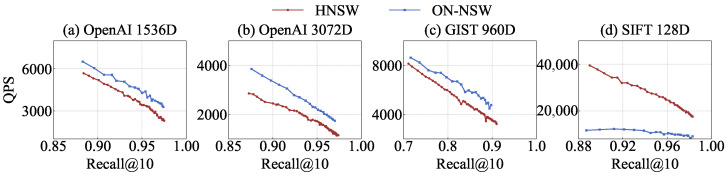
Throughput comparison of the original HNSW and ON-NSW with respect to Recall@10.

As shown in Figure 4a–c, ON-NSW outperforms the original HNSW on high-dimensional vector datasets, achieving 1.44×, 1.41×, and 1.42× higher throughput on OpenAI and GIST, respectively, at comparable recall levels. This performance gain stems from ON-NSW ’s ability to exploit GPU parallelism to compute distances for high-dimensional vectors and to process queries in batches across blocks. Within each block, ON-NSW leverages warp-level execution to compute the distances of candidate nodes in parallel.

Furthermore, the original HNSW performs distance calculations sequentially during a search operation, which results in many hops. In contrast, ON-NSW evaluates multiple neighbor nodes in parallel within a single hop, thereby completing the search with fewer hops. For the OpenAI dataset with 1536 dimensions, the original HNSW performs an average of 79 distance computations in the upper layers before reaching layer 0. In layer 0, it requires about 33 hops, with 16 distance computations per hop. By comparison, ON-NSW starts the search directly from a fixed entry point in layer 0 and requires only 7 hops on average, with about 193 distance computations per hop. Although the number of distance computations per hop is higher in ON-NSW, its use of GPU warp-level parallelism leads to significantly better search performance than the original HNSW.

Because ON-NSW removes the upper hierarchy of HNSW to maximize GPU parallelism, the retrieved results are not guaranteed to be exactly the same as those of the hierarchical algorithm. However, the result quality is controlled by the recall parameter, ensuring comparable accuracy between ON-NSW and the original HNSW. Unless otherwise specified, all experiments were conducted under the same recall level, providing a fair comparison of throughput and resource efficiency.

As shown in Figure 4d, ON-NSW exhibits up to 2.8× lower throughput than the original HNSW at comparable recall levels. This is because the execution time of distance computations is relatively short for low-dimensional data, which limits the benefit of GPU parallelism. For such datasets, distance calculations can be efficiently handled on CPUs within a short time. In addition, ON-NSW requires an extra sorting step after distance computations, whose impact becomes more pronounced for low-dimensional data. For the SIFT dataset, only about 14% of the total search time is spent on distance computations, whereas more than 60% is consumed by sorting. In contrast, the original HNSW does not require sorting, and distance computations can be efficiently performed on CPUs, resulting in higher search throughput than ON-NSW.

**Figure 5:**
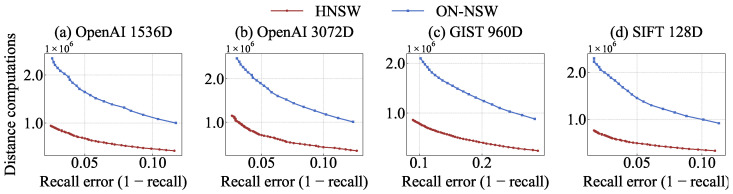
Distance computation comparison of the original HNSW and ON-NSW with respect to Recall@10 error.

**Total Execution Time.** ON-NSW consistently outperforms the original HNSW on high-dimensional datasets across all Recall@10 levels. For example, at Recall@10 values of 0.974 and 0.971 on the OpenAI 1536D and 3072D datasets, respectively, ON-NSW reduces the total execution time by about 27% compared to HNSW (0.305 s vs. 0.417 s). A similar trend is observed on the GIST dataset, where at a Recall@10 of 0.900, ON-NSW completes in 0.210 s while HNSW requires 0.280 s. In contrast, on the relatively low-dimensional SIFT dataset, at a Recall@10 of 0.981, HNSW remains faster (0.054 s vs. 0.103 s).
**Distance Calculation Comparison.** As shown in Figure 5a–d, ON-NSW performs up to 3.5× more distance computations than the original HNSW at the same recall level. This occurs because ON-NSW exploits GPU parallelism to compute distances for a larger number of vector data within each hop. Although the total number of computations increases, ON-NSW reaches vectors closer to the query more quickly by fully utilizing GPU parallelism.

### 4.3. Throughput over Varying Number of GPU Blocks

We compare the throughput while varying the number of GPU blocks. Each block consists of 1024 threads, which is the maximum supported per block in ON-NSW. Recall is measured as Recall@10.

**Figure 6:**
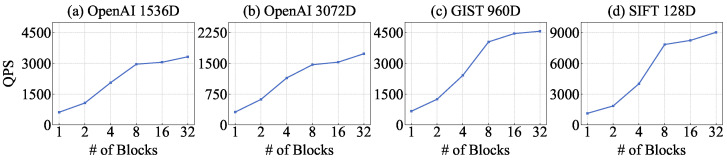
Throughput comparison of ON-NSW across different numbers of GPU blocks (#, number of blocks) at Recall@10.

As shown in Figure 6a–d, throughput increases with the number of GPU blocks across all datasets. However, the performance improvement from 8 to 32 blocks is smaller than that from 1 to 8 blocks because the maximum number of concurrently active blocks is constrained by hardware resources such as registers and shared memory. ON-NSW uses 32 blocks as the optimal configuration for all subsequent experiments.

Under the current kernel configuration, only two blocks can be active per SM, allowing up to 16 blocks to execute in parallel across the GPU. When more than 16 blocks are launched, the excess blocks remain pending in the scheduling queue until resources are released. Moreover, with 16 active blocks, the number of warps is already sufficient to hide the memory latency of DRAM accesses for vector data loading. Therefore, increasing the number of blocks only raises the proportion of stalled warps without providing additional performance gains.

### 4.4. Performance Breakdown

We analyze the execution time of each search step in the original HNSW and ON-NSW across all datasets. The original HNSW traverses the upper layers, identifies the nearest neighbors in layer 0, and maps the internal node IDs to the external labels. In contrast, ON-NSW copies the query vector directly to the GPU kernel instead of traversing the upper layers, while the remaining steps are the same. The original HNSW executes the search using six threads, whereas ON-NSW launches a kernel with 32 blocks of 1024 threads and then employs six threads for label mapping. The search parameter *ef* is set to the maximum value of 30 in ON-NSW and adjusted in the original HNSW to achieve the same Recall@10.

**Figure 7:**
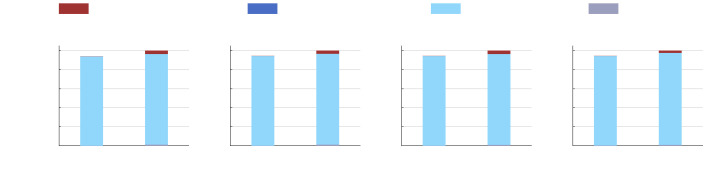
Breakdown of search execution time for the original HNSW and ON-NSW.

As shown in Figure 7a–d, about 95% of the execution time is spent on the layer 0 search for both the original HNSW and ON-NSW. This is because distance calculations between vector data are repeatedly performed, dominating the overall search time. In addition, data accesses to neighbor nodes are mostly random accesses, which causes frequent cache misses and results in longer execution times.

For all datasets, only about 3.5% of the execution time in ON-NSW is spent on copying query vectors to the GPU. This is because ON-NSW reduces the number of cudaMemcpy() calls by processing queries in batches. The original HNSW requires about 5.5% of the execution time for upper-layer traversal, whereas ON-NSW incurs no such cost since it does not perform this step. For label mapping, the original HNSW accounts for 0.16% of the execution time, while ON-NSW requires about 1%. Because ON-NSW stores the top-k node IDs and distances in a buffer allocated in pinned memory, which has higher latency than DRAM, it introduces additional overhead. Nevertheless, its impact on the overall execution time is negligible.

### 4.5. Energy Efficiency

We compare the power consumption of ON-NSW and the original HNSW during ANNS execution. Power was measured using NVIDIA’s tegrastats tool (included in JetPack 5.1.4, L4T R35.5.0, Feb 2024), which samples instantaneous power every 100 ms. For each dataset, we set the same Recall@10 level for both HNSW and ON-NSW and measured the corresponding power consumption. Specifically, the Recall@10 level was fixed to the value used for the total execution time analysis in Section 4.2. We executed 1000 queries ten times and calculated the average power consumption over all runs. Similar to the previous experiments, HNSW used 6 CPU threads, while ON-NSW utilized 32 GPU blocks, each consisting of 1024 threads.

**Figure 8:**
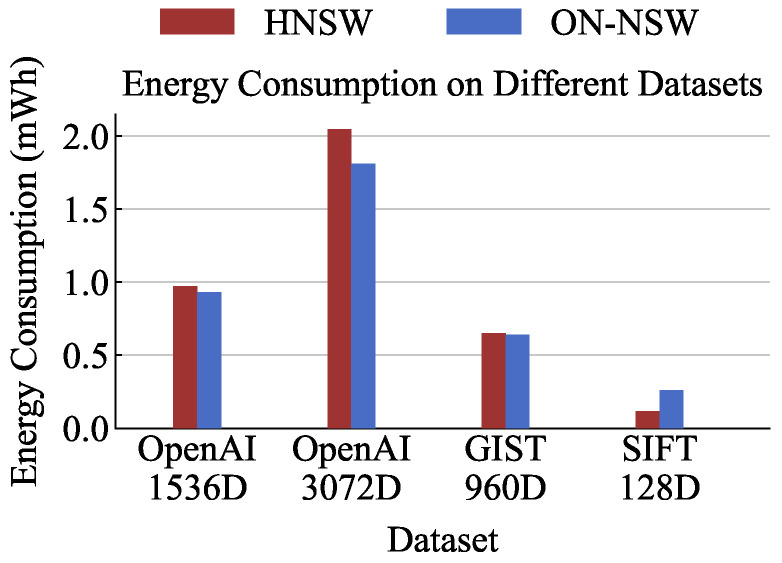
Energy consumption comparison over different datasets for the original HNSW and ON-NSW.

As shown in Figure 8, ON-NSW achieves up to 13% lower power consumption than the original HNSW on high-dimensional datasets. Although the GPU contains thousands of CUDA cores and therefore exhibits higher instantaneous power draw than the CPU, ON-NSW completes ANNS in a much shorter time, resulting in lower overall power consumption. In contrast, for the low-dimensional SIFT dataset, ON-NSW shows approximately twice the power consumption of HNSW.

### 4.6. GPU Utilization on an Edge Device

We analyze the GPU resource utilization of ON-NSW during ANNS using the Roofline model. For this experiment, we use the OpenAI 1536D dataset and execute 1000 queries with 32 GPU blocks, each consisting of 1024 threads. The theoretical peak performance of the GPU is 1.28 TFLOP/s, calculated from the number of CUDA cores (1024), the GPU clock frequency (625 MHz), and the FP32 throughput of 2 FLOPs per cycle. The theoretical memory bandwidth is 68 GB/s [37].

**Figure 9:**
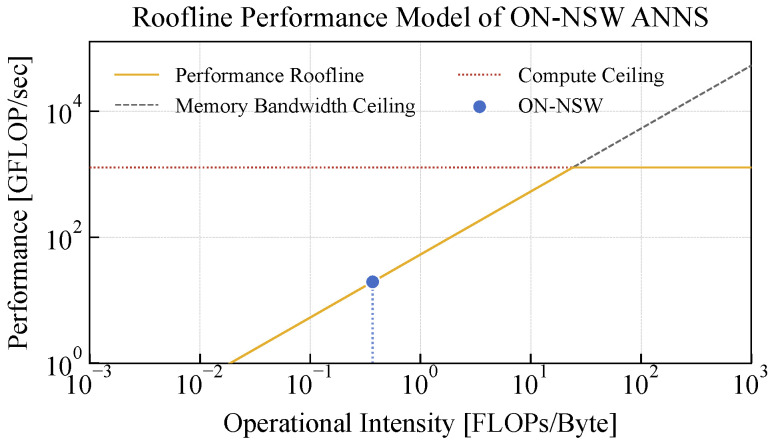
Roofline model illustrating the GPU utilization of ON-NSW during ANNS.

As shown in Figure 9, the GPU utilization of ON-NSW during ANNS is limited by a memory access latency because the vector data are stored in pinned memory, which introduces high latency. The SM Busy metric, representing the average SM utilization, is 22%, indicating that approximately 78% of the SMs are stalled waiting for memory accesses. Furthermore, since most data accesses in ANNS are random, ON-NSW exhibits a low cache hit rate. The hit rates of the GPU L1 and L2 caches are 41% and 1.8%, respectively, indicating that most data accesses are served directly from main memory.

## 5. Related Works

### 5.1. GPU-Accelerated ANN Search

Recently, several studies have investigated GPU acceleration for ANNS queries. SONG [38] improves query performance by reducing GPU memory allocations and memory consumption. CAGRA [39] accelerates both graph construction and query processing by exploiting GPU parallelism. ParaGraph [40] employs CPU–GPU co-processing for efficient graph construction and cross-modal ANNS. Tagore [41] proposes an asynchronous CPU–GPU–disk framework to mitigate performance bottlenecks caused by coordination overhead. FAISS [42] also provides GPU implementations of ANNS algorithms, including HNSW.

While these efforts demonstrate the benefits of GPU acceleration, they mainly focus on high-end servers equipped with discrete GPUs. In contrast, edge platforms such as NVIDIA Jetson devices feature unique architectural characteristics, notably a unified CPU–GPU memory hierarchy, which are rarely considered in existing work. A recent study [43] evaluated ANNS on edge devices, but primarily by porting existing algorithms without consideration for architectural characteristics. ON-NSW redesigns HNSW specifically for edge GPUs, maximizing parallelism while fully addressing the distinct characteristics of embedded platforms.

### 5.2. HNSW Variants

Graph-based Approximate Nearest Neighbor Search (ANNS) methods have attracted significant attention due to their high recall and efficiency. Among them, HNSW has inspired a variety of extensions. Dynamic HNSW [44] improves performance by dynamically optimizing index parameters. HNSW++ [25] introduces a dual-branch design to accelerate ANNS. d-HNSW [45] adapts HNSW to disaggregated memory systems with an RDMA-friendly layout and caching. DIGRA [46] integrates multi-way tree structures with NSW graphs to support range-filtering queries.

While these studies show that HNSW can be extended for various purpose, they primarily target CPUs in high-end servers. In contrast, ON-NSW redesigns HNSW for GPU on edge devices, with a focus on parallelism and memory hierarchy optimization.

## 6. Conclusions

In this work, we presented ON-NSW, a GPU-centric variant of NSW that redesigns HNSW for efficient execution on edge devices. ON-NSW flattens the hierarchical structure of HNSW to fully exploit GPU parallelism and optimizes memory placement to take advantage of the unified CPU–GPU memory architecture of NVIDIA Jetson devices. Our evaluation demonstrates that ON-NSW significantly improves the throughput of HNSW for high-dimensional datasets while maintaining comparable recall.

As future work, we plan to extend the proposed design and optimizations to a broader range of edge platforms and emerging hardware architectures. In addition, we aim to develop an embedded vector database system based on ON-NSW to further validate its practicality in real-world deployments. Another important direction is to enhance the performance of ON-NSW on low-dimensional datasets, where GPU parallelism is less effectively utilized, by exploring new search strategies and memory management techniques.

## Data Availability

Data are contained within the article.
